# Layers of inequality: gender, medicalisation and obstetric violence in Ghana

**DOI:** 10.1186/s12939-024-02331-z

**Published:** 2024-11-20

**Authors:** Abena Asefuaba Yalley

**Affiliations:** Department of Politics and Public Administration/Zukunftskolleg, University of Konstanz, Konstanz, 78464 Germany

**Keywords:** Obstetric violence, Gender inequality, Patriarchy, Systemic injustice, Health systems, Childbirth, Ghana

## Abstract

**Background:**

This study explored how gender inequalities in health systems influence women’s experiences of obstetric violence in Ghana. Obstetric violence is recognised as a major public health concern and human rights violation. In particular, it reduces women’s trust and use of health facilities for childbirth, thereby increasing the risks of maternal and neonatal mortality. In Ghana, obstetric violence is pervasive and normalised; yet, little is known about the gendered dynamics of this phenomenon.

**Methodology:**

A qualitative study was conducted in eight public health facilities in Ghana. Specifically, semi-structured interviews were conducted with 30 midwives who work in the maternity units and 35 women who have utilised the obstetric services of the hospitals for childbirth. The midwives and women were selected using the purposive sampling technique. The transcripts of the interviews were coded using NVivo qualitative data analysis software and were thematically analysed. Secondary materials such as existing data on the medical profession in Ghana were utilised to complement the primary data.

**Results:**

The study revealed that there are huge structural inequalities that keep women at the lower cadres of the health system. Five major themes depicting how gender inequalities contribute to women’s experiences of obstetric violence emerged: gender inequality in the medical profession, unequally and heavily tasked, feminisation of midwifery, patriarchal pressures and ideologies, and gender insensitivity in resource provision. These inequalities impact the kind of care midwives provide, which is often characterised by mistreatment and abuse of women during childbirth. The study also discovered that patriarchal ideologies about women and their bodies lead to power and control in the delivery room and violence has become a major instrument of domination and control.

**Conclusion:**

The hierarchical structure of the healthcare profession puts the midwifery profession in a vulnerable position, with negative consequences for maternity care (obstetric violence). The study recommends that gender-responsive approaches that address structural inequalities in health systems, women’s empowerment over their bodies and male involvement in women’s reproductive care are crucial in dealing with obstetric violence in Ghana.

## Background

Violence and abuse of women during childbirth is a critical and pressing public health concern. Complaints of women about poor and abusive treatment during childbirth in health facilities are emerging in global health discussions [1–3]. The literature documents situations where women are exposed to physical violence in the form of beatings, violent vaginal examinations, forced episiotomies, coercion, unnecessary medical interventions, including denial of treatment, and lack of informed consent. Long before the concept of obstetric violence was coined in Latin America, feminists had contended against the dehumanisation of women during childbirth in the second wave of feminism in the 1970s [4, 5]. However, the phenomenon itself only began to receive wider attention in the mid-2000s due to the work of activists, who criticised the over-medicalisation of childbirth and called for humanised birthing in Latin America.

Since then, several studies have demonstrated that violence during childbirth is widespread, threatening the lives of many women worldwide [2, 6, 7]. Vacaflor [8] clearly defines obstetric violence as “the violence exercised by health personnel on the body and reproductive processes of women (during pregnancy or delivery), as expressed through dehumanising treatment, medicalisation abuse, and the conversion of natural processes of reproduction into pathological ones”. Obstetric violence as a concept is relatively new in the global health literature with researchers adopting various terminologies such as “disrespect and abuse (D&A)”, “mistreatment and abuse”, “dehumanised childbirth” and “disrespectful maternity care” to describe violence and abuse in obstetric care. While these terminologies help to categorize the different manifestations of violence, the term “obstetric violence” emphasizes the “structural dimensions as a gender-based violence that intersects with institutional violence” [3, 4]. Nonetheless, all the terminologies acknowledge the harmful effects of violence, the dehumanisation of childbirth and the violation of women’s rights and dignity.

In this study, the term “obstetric violence” is used interchangeably with the other terminologies. According to the World Health Organization (WHO) [3], obstetric violence is classified into “physical violence, humiliation and verbal abuse, intimidation, forced medical procedures, neglect, lack of confidentiality, failure to seek consent, refusal to administer pain medications, avoidable complications, refusal of medical admission, and detention of women after delivery based on their inability to pay medical bills”. Although the global prevalence of this phenomenon is unknown, studies reveal that violence during childbirth is widespread in both high, middle and low-income countries. Obstetric violence rates range from 17% in the USA, 44% in Argentina, 15% in India, 38% in Spain, 76% in Turkey, 20% in Kenya to 78% in Ethiopia [9–15]. The immediate consequences of obstetric violence for women’s health include increased risk of caesarean sections and complications such as vaginal trauma, postpartum haemorrhage, physical lesions as well as psychological trauma [16, 17]. In the long term, traumatic memories of labour may limit the desire for a new pregnancy or reduce satisfaction and trust in healthcare professionals [17–19]. Corea et al. [10] revealed in their studies that in Latin America, obstetric violence is the most cited reason for women’s refusal to use facility-based services for childbirth, leading to a substantial increase in maternal mortality.

In Ghana, maternal mortality and morbidity continue to be a major public health concern. Recent data has revealed that 263 Ghanaian women die in every 100,000 live births [20]. These figures are still far above the global maternal mortality target of 70 deaths per 100,000 live births (Sustainable Development Goals 5). Research has demonstrated that the majority of these deaths can be prevented by providing quality maternal care and obstetric services [21]. Up to 70% of maternal deaths could be prevented if all deliveries were conducted by skilled birth attendants [21, 22], making Skilled Birth Attendance a major cornerstone to reducing maternal mortality. However, more than one-third of women (35%) (out of 95% who attend antenatal care services) do not utilise skilled birth attendants for childbirth due to poor quality care [23, 24]. Obstetric violence is pervasive in Ghana, with 65% of women stating that they have been victims of it [25]. Women’s experiences include brutal acts of physical violence, abandonment, verbal abuse, lack of privacy, unconsented care and stitching without anaesthesia [2, 26, 27]. Single mothers, teenagers and HIV-positive women are at a higher risk of being mistreated [25, 27]. Sometimes women are even abandoned to deliver without the assistance of healthcare personnel. Women avoid skilled birth attendants if they believe they will be abused, thereby contributing to the high maternal mortality in Ghana [28, 29]. This pervasive abuse in the context of childbirth has raised concerns about the safety of women in the delivery room and the WHO has called for increased research on the issue, particularly investigations into the key drivers of obstetric violence [2].

Without doubt, there is a deeply embedded culture of violence in the delivery room and understanding the dynamics of this culture, the drivers, and how it is sustained is critical to dealing with the problem of obstetric violence. This paper argues that obstetric violence is deeply entrenched in structural inequalities, that it is a by-product of gender inequalities and a form of patriarchal violence within society and health systems. As patriarchy depends upon institutions for its sustenance, the healthcare system can itself become an agent for patriarchal performance. In addition, because childbirth is ascribed to femininity, obstetric care can be impacted by gender inequalities. In patriarchal societies like Ghana, healthcare professionals are likely to uphold gender stereotypical ideologies which could compromise how women are perceived and cared for. Childbirth particularly puts a healthy woman in a fragile space, restricting their autonomy over their bodies and making them especially vulnerable to established social norms [30]. Shabot [31] contends that “obstetric violence is different from other forms of medical violence because labouring and birthing bodies are not ill, diseased, or dysfunctional, rather the labouring body is a healthy and powerful body”. Hence, the idea of a medicalised childbirth is an androcentric ideology that aims to domesticate women’s bodies and keep women in subjection.

Again, the healthcare profession is feminised, with women constituting more than 70% of the health workforce in Ghana [32]. While this could be interpreted as an advantage for women, there is rather a very skewed representation across the higher levels of the medical profession. Women comprise about 35% of all medical doctors; while almost 86% of nurses and midwives are women [32, 33]. This has a greater impact on women’s representation in the decision-making bodies since higher-level personnel constitute the bodies. Unequivocally, men dominate the governing bodies and high-level councils where crucial decisions regarding the administration of the health systems and policies are taken. This creates structural inequalities that still put women in a subjugated position in the medical profession and the health system as a whole. Previous studies have established that midwives and nurses in Ghana work in an uninhabitable environment clouded by scarcity of resources, “oppressive conditions” and dominated by “workplace violence” [34]. Overall, this is also likely to compromise the quality of healthcare delivered to women.

From the foregoing, it is an indisputable fact that obstetric violence is a feminist issue, which must be examined from a feminist perspective. Yet none of the studies on obstetric violence in Ghana have investigated the gendered dynamics of obstetric violence, particularly how the societal conceptualisation of women is replicated in healthcare systems and how stereotypical gender norms influence care. This study specifically interrogates gender inequalities in health systems and explores how they contribute to women’s experiences of obstetric violence. This is crucial to understanding how gender inequality drives obstetric violence, providing a timely contribution to designing impactful interventions that can help to confront the primary causes of obstetric violence in Ghana.

## Methods

### Study design and setting

This study was conducted in the Western and Ashanti Regions of Ghana using the feminist phenomenological research approach. Feminist phenomenology offers a critical approach for exploring particular phenomena from a gendered perspective [35]. Because childbirth experiences are influenced by cultural and institutional contexts, this critical qualitative approach enables a comprehensive analysis that focuses on gendered embodiment and how gender impacts one’s experiences and understandings of socio-political systems and institutions [35]. Data was collected in eight public health facilities in both urban and rural settings. Specifically, empirical data was collected in the Kwesimintsim Polyclinic and Essikado Government Hospital (urban), and the Agona Nkwanta Health Centre and Dixcove Government Hospital (rural) in the Western Region. In the Ashanti Region, the study was conducted in two healthcare facilities located in the Kumasi Metropolis (urban) – the Maternal and Child Hospital and the Tafo Government Hospital – and two hospitals serving the rural communities in the Ashanti Region – Nkenkaasu Government Hospital and Ejura District Hospital.

### Study population and sampling

Semi-structured interviews were conducted with women and midwives in the respective hospitals. Interviews are particularly appropriate for exploring a specific phenomenon thoroughly and for acquiring comprehensive information regarding personal experiences and perspectives [36]. In Ghana, midwives conduct 80% of all institutional deliveries [37], making their views germane to this study. The purposive sampling technique was employed to select 30 junior and senior midwives in the maternity wards for the interviews. All midwives who participated in the study were licensed practitioners who had conducted deliveries in the past and were at different stages of their careers – junior, senior and principal midwives. Overall, the majority of the midwives [20] worked in urban health facilities while 10 worked in rural health facilities. In addition, 35 women who had accessed the obstetric services of the hospitals for childbirth in the past 24 months were purposefully selected and interviewed. The women were between the ages of 15 and 40 and resided in the Western and Ashanti Regions. The sample size was guided by the principle of saturation – a qualitative research technique whereby the researcher ceases gathering data because no new information emerges from subsequent interviews [38]. Therefore, the sample size of this study was considered sufficient for interrogating the gender dynamics of obstetric violence.

### Data collection procedure

Prior to the interviews with the midwives, a meeting was held with the hospital administrators, where the rationale of the study and the ethical approval letter were presented for their consent. Thereafter, the chief midwives in the maternity wards were first approached, and midwives who were on duty and willing to participate in the study were interviewed. Even though most midwives were keen to participate, a small number declined to participate due to time constraints. A semi-structured interview guide was used to collect data on their professional experiences as midwives and obstetric violence. The questions on obstetric violence were based on Bowser and Hill’s seven performance indicators of obstetric violence – physical abuse, non-consented care, non-confidential care, non-dignified care, discrimination, abandonment of care, and detention in facilities [39]. Thereafter, women who had delivered in the health facilities were approached at the immunisation clinics where they brought their children for immunisation. The rationale for the study was adequately explained to them and women who consented to participate were interviewed in their preferred locations. The interviews explored women’s childbirth experiences in health facilities with particular emphasis on their experiences of obstetric violence as women. The interviews with the women were mostly conducted in Akan, while, most midwives preferred to be interviewed in English. Data were audio recorded upon consent from participants. In addition to the primary data, secondary data sources such as existing public information on the medical profession in Ghana was utilised. The interviews were conducted between August 2021 and February 2022. As a young Ghanaian mother with childbirth experiences in Ghana, the researcher was conscious of how her personality, notions and experiences could influence the data. To deal with these potential biases, the researcher utilised diverse data collection approaches and sources, such as semi-structured in-depth interviews with mothers and healthcare personnel as well as documented sources to ensure objectivity. The rigour of the research findings was established through reflexivity, transferability and audit trial. The utilization of the purposive sampling method ensured that only interviewees who possessed the required experience and knowledge were interviewed. Again, the use of diverse data sources – interviews with midwives and women, supplemented by secondary materials – promoted objectivity. To promote transferability and replication of the study in the future, the protocol of the research was strictly followed and the research methodology and procedure are robustly described.

### Data analysis

All the recorded interviews were transcribed and analysed according to Braun and Clarke’s [40] framework for conducting thematic analysis. This involves the identification of patterns and emerging themes from the qualitative data to answer research questions. Following Braun and Clarke’s Framework, the transcribed interviews were first meticulously read for familiarity and to check for inconsistencies. Thereafter, the data was exported into NVivo Qualitative Data Analysis Software, Version 12 where codes and sub-codes were generated using the inductive approach-repetitive reading of the data, in order to identify common or emerging themes. For the purpose of this paper, only codes that focused on gender and obstetric violence were extracted and analysed. The codes were further reviewed for major themes and sub-themes. Data from the secondary sources were extracted manually and analysed in a similar manner to complement the primary data.

### Ethical considerations

This study was approved by the Ethics Committee of the University of Konstanz, Germany (IRB Statement 37/2021) and the Ghana Health Service Ethics Review Committee (GHS-ERC 010/06/21). In addition, the directors of medical services in all the health facilities granted their consent. Individual consent was sought from all the women and midwives who participated in the study. This is a sensitive research subject, requiring strong confidentiality and anonymity. To ensure confidentiality, the names and telephone numbers of the participants were not collected. Also, the health facilities of the participants are concealed in this report to safeguard their identity as some health facilities (especially in rural settings) have few midwives (sometimes only two); hence, they could be easily traced.

## Results

### Major themes and sub-themes

**Fig. 1:**
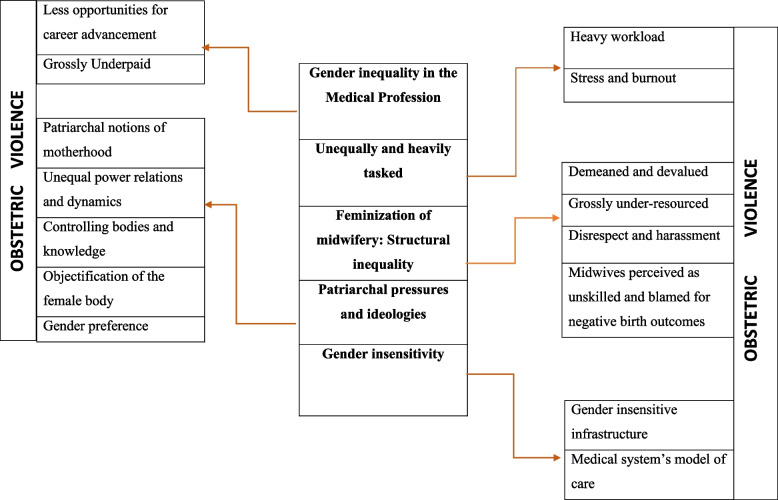
Major themes and sub-themes on gender inequality in health systems and obstetric violence

This study explored how gender inequalities in health systems influence women’s experiences of obstetric violence in Ghana. Five conceptual themes with diverse sub-themes inductively drawn from the primary and secondary data are presented in Fig. 1.

### Gender inequality in the medical profession

Gender inequality in health systems manifests itself in terms of representation and power dynamics, conditions of service and opportunities for professional advancement. Women occupy the lower cadre of the profession and have limited decision-making powers and autonomy. Midwives revealed that these hierarchical power dynamics influence their careers, consequently affects the kind of care they provide.

#### Less opportunity for career advancement

Particularly, midwives lack career advancement opportunities and in-service training, mainly on contemporary delivery methods and skills. Especially for older midwives who received their training over 30 years ago, there is a wide knowledge gap. The following extracts from the midwives’ interviews stress the link between in-service training and quality care:

*I think education is our problem! Education!! There is no in-service training, so what you knew yesterday is the only knowledge you have. Therefore, if new things [skills] come and you are not trained, you remain archaic. That’s it. So, if there is a new skill to support mother and baby and you don’t know it, definitely it may lead to it [obstetric violence]. (Midwife 9)*

*Schooling; furthering of education is challenging. In the sense that when you complete school and begin working, the midwife will not be allowed to go and seek more knowledge, fresh knowledge to improve the work, including workshops. If they will not allow us to go to school, we are also there using our archaic knowledge. (Midwife 19)*

Sometimes the midwives lacked the technical know-how, thereby often resulting in the use of violence to ensure a successful delivery. When midwives were asked about their opinion on obstetric violence, some noted that mistreatment during childbirth is wrong but they ‘have no other alternative' for a violence-free delivery. For example, women’s right to their preferred birth position is sometimes denied because of perceived notions that the lithotomy position is the safest.

*When the woman has dilated but is not pushing, I will first shout, then beat the tighs. If she doesn’t push, I will cut her vagina. This puts fear in them to push and they will think it is abuse but I am doing the right thing to have a successful delivery. (Midwife 5)*

*Yeah, we have different birth positions, but here we are all using the lithotomy position because labour comes with many complications. If the baby is coming with other parts of the body and the woman is fat, imagine how difficult it’s going to be. So, the lithotomy position is used to manage complications. (Midwife 2)*

*Some [women] will prefer squatting, and some will even prefer the doggy style position, but the Fulani women normally use those styles because most of their deliveries are without support. But here someone is delivering you so they position you in that form. It is easier. (Midwife 10)*

#### Grosslyunderpaid

The unequal power relations also manifest in employment conditions and benefits for female caregivers. The findings of this study revealed that midwives are grossly underpaid with an average monthly income of ₵2,000 ($138). This constitutes less than 30% of the average salary of medical doctors (₵7,000, equivalent to $476) in Ghana. Many midwives therefore engage in alternative trading activities in the maternity wards to supplement their poor salaries. Some of the items sold include baby diapers, clothes, water, and sometimes food. The interviews revealed that women are sometimes neglected or abandoned because midwives focus more on trading activities. In addition, there is evidence of the detention of women who are unable to pay for items purchased. During an interview with a midwife, she noted:

*If they are not able to pay? You [women] will be able to pay, how won’t you be able to pay? Can you go to the market to collect things and say that you won’t be able to pay? You will be able to pay. If you don’t give me the money, I won’t give you the items and you will be there. We have detained several women. We have a reason why we are keeping them here and no one can come and tell us otherwise. They bought something from us but did not pay. (Midwife 7).*

Both midwives and women admitted that there is a lot of unauthorized extortion by midwives (both items and cash), which often induces discrimination and favouritism in care. The following are narrations from both midwives and women:

*If you treat the client well and they are leaving they can say, take this money but some ask the client and they term it “egg money”. (Midwife 27)*

*It makes them treat the woman right, only when the husband gives them a tip but don’t treat a woman right if her husband doesn’t tip them. (Mother 9)*

*After delivery, when we were about to leave, they took one toilet roll, three soaps and 100 Ghana cedis. (Mother 28)*

*That’s how they treated all of us. There was even one lady who when she got there, the midwife told her that she should pay for the bedsheet they provided. And she was even saying that we should pay for the light but the lady also protested and it became an argument. One of the mothers said she should just pay, otherwise, maybe they would not allow her to take her child home and she paid. (Mother 5)*

### Unequally and heavily tasked

The results indicate that midwives are much more heavily and unequally tasked. This was revealed both in the interviews conducted and existing data on the medical profession in Ghana.

#### Heavy workload

While workload is generally a problem for all health workers, there is an uneven distribution across the female-dominated profession. In Ghana, the midwife-patient ratio is 1 per 560 patients while the doctor-patient ratio is 1 per 6,355 patients [41]. When analysed according to WHO recommendations [42, 43], the midwife-patient ratio is 140 times higher than the recommended ratio (1:4), while the doctor-patient ratio is about 6.4 times higher (1:1000). Particularly for the midwifery profession, this is problematic as it is inherently dangerous or simply impossible to conduct multiple deliveries at the same time. The midwives interviewed revealed their heavy workload contributes to the mistreatment of women during childbirth.

*Too much workload can lead to that – when you have a shortage of staff and there’s too much workload. When we are attending to multiple women at the same time, at times we neglect the ones who are not that serious. (Midwife 14)*

*Some of the clients disturb a lot. It’s true. When we have so many clients to attend to and we see a particular person who is disturbing, we will ignore you totally. (Midwife 29)*

*Somebody (midwife) can come to night duty and deliver like 20 babies or even imagine 10 babies, the midwife will be roaming helter-skelter and if you [woman in labour] misbehave, the midwife will shout at you unintentionally. (Midwife 11)*

#### Stress and burnout

The midwives contended that excessive workload leads to stress and burnout. This causes tensions, fatigue and sometimes anger that is subsequently unleashed on the labouring woman. The following interviews reveal how stress and burnout lead to obstetric violence:

*It's because of the stress we go through to care for all the patients. Sometimes it's a transfer of anger. Imagine all the beds being full and one person who is not having contractions or progressing is complaining about the pain and giving false alarms. That one the client will be ignored, you get me? (Midwife 7)*

*You [midwife] are posted to a place where we have a lot of women coming there to deliver. Maybe this person is 10 cm and the other has the head of the baby in the vagina, I want this one to push very fast to deliver so that I will go to the other person. When the person is not doing it, definitely I will shout*. *(Midwife 16)*

*Midwives also need sound minds to do the work. Out of anger, a person [midwife] can harm because when a person is angry the brain is out of work. (Midwife1)*

The choice of a preferred position for a comfortable and easier childbirth is a fundamental right of all women. Although this right was acknowledged by the midwives who were interviewed, women are sometimes restricted to the lithotomy position because of the stress. Indeed, some midwives even admitted that some birth positions such as squatting were easier for women but they outrightly denied these options. A midwife, narrating her experience disclosed that:

*The women cannot choose which position they want to deliver, no. We tell them to lie in the lithotomy position. The squatting position is distressing and stressful for us although I learned that it is easier for women to deliver squatting than the lithotomy. (Midwife 18)*

Another midwife emphasized:

*A woman during delivery insisted on squatting and it was hell for us. It was during a night shift and only two midwives were on duty. I remember the other midwife had to lie at her back and I lied in front to see if the baby was coming and to receive the baby. There was blood all over the place and we ourselves were soaked in blood. It was a bad encounter and I would not wish to experience that again. (Midwife 11)*

From the above quotes, it is obvious that midwives avoid other birth positions due to stress and burnout. With crowded maternity wards with pressure to conduct multiple deliveries, denying women access to their preferred birth position is a way of dealing with this stress and burnout.

### Feminisation of midwifery: Structural inequality

When investigating how gender constructions play a role in women’s experiences of obstetric violence, one major theme that stands out is the feminisation of the midwifery profession, virtually synonymous with women. Midwifery is viewed as an extension of women’s traditional care and nurturing roles in society, and therefore, subjected to the traditional conceptualization of women as second-class citizens. Furthermore, the socially constructed reproductive role of women puts the profession in an even more vulnerable and subjugated position, one of inferiority and reduced importance within the healthcare system.

#### Demeaned and devalued

Midwives felt demeaned, devalued and disrespected, which undermined their efforts. This sometimes leads to a transfer of aggression, resulting in poor care and mistreatment of women. The extracts from the interviews below demonstrate how the poor treatment of midwives lead to the mistreatment of women:

*Like when you are doing something and you get it wrong, they [authorities] should not tell you in front of clients [women], they should excuse you, so that the client will not go and say it somewhere. (Midwife 17)*

*You are giving your best and somebody [those in authority] is somewhere trying to undermine whatever you are doing; you are human and definitely you will get annoyed somehow and if the client [woman] doesn’t take care, you transfer aggression. (Midwife 7)*

*My expectation from my supervisors is that they should teach us the upcoming ones some of the skills and correct us with love. They sometimes insult us or correct us rudely before the patients. (Midwife 14)*

#### Grossly under-resourced

As a feminised profession, the entire midwifery sector is grossly under-resourced and has poor working conditions compared to other medical sectors. Midwives complained of neglect regarding the basic tools and equipment needed for the job. These include vacuum extractors, sterilizers, cardiotocography, surgical gloves, etc. They either have to purchase the equipment from their meagre salaries or are forced to work without them. These factors compound to trigger abusive behaviour. Women are sometimes abandoned or denied treatment due to a lack of resources.

*Let’s say you are for a weekend shift and you have a shortage of logistics and supplies, how would you attend to the patient? Let’s say the patient is HIV positive, do you expect the midwife to go and use her bare hands to conduct a delivery? (Midwife 25)*

*There were no gloves for me to use, so imagine if she [the woman] was an HIV patient, she would have infected me. I used my bare hands. I lifted the baby and released the cord from the neck. I made the baby survive. But the doctor came and blamed me. He did not appreciate my effort but said if I did not have gloves, I should have allowed the baby to slide and fall. (Midwife 16)*

*All of them, sometimes the room is very hot you will be sweating and all your dress is wet, this woman [labouring women] will not do faster for you to move out*. (Midwife 9)

*When women come to the hospital you have to make sure all the instruments are sterilized for zero percent infections. But our autoclaves are spoilt so we use a gas that we use at home to boil the instruments, so imagine the infection prevention rate will be high. (Midwife 22)*

#### Disrespect and harassment

Midwives complained about a general lack of respect for the profession. They experienced disrespect not only from other health professionals, but also from members of the public. Sometimes, they are even harassed by relatives of the women they are caring for, which triggers provocation and can lead to poor treatment of women. To exert the recognition and respect they are often deprived of, midwives engage in disrespectful behaviour to maintain power and control over women. Recounting their experiences, midwives noted:

*One day come and work with us and you will see that midwives are doing very well. Some people, their relatives will come and curse. They will come and be dictating to us and all sorts of things. (Midwife11)*

*Last month, one woman came here for labour but bled after delivery and because of this, she had to undergo a blood transfusion. While we were waiting for her laboratory results, her relative came to complain about how she had been in the hospital for so long and requested that we discharge her. The relative was rude to the midwives and I had to speak some sense into her. (Midwife 7)*

*A person [woman] comes to deliver and we have given her treatment but no delivery on the second day. The relatives come here to insult us that we have admitted the wife and she has not delivered until now. When that one happens, at least we have to let them know that we are not just sitting here ooh, we are also working. Therefore, if I admit your relative and am treating her, we are still on our treatment and you come here to insult us, we will not take it. (Midwife 6)*

#### Midwives are perceived as unskilled and blamed for negative birth outcomes

The midwives are also viewed as unskilled and blamed for every negative delivery outcome. During this process (termed ‘auditing*’*), a midwife’s license could be revoked, causing her to lose her job and livelihood. This places increased pressure on them. Midwives used keywords such as *fear*, *frustration, anxiety* and *trouble* to describe their pervading work situation. The labouring woman, in this sense, is perceived as either a facilitator or barrier to the career success of the midwives. In a bid to avoid auditing, midwives employ every means, including violence to force successful delivery. The following extracts from the data buttress how auditing leads to obstetric violence.

*When the baby or the woman dies, the midwife is held responsible because pregnancy is not a disease. Because a midwife doesn’t want any problems, she will beat the woman so that the woman can push so that she won’t have any problems. (Midwife 10)*

*I have an experience, a midwife hit a woman to cause her to push. This is because some of the clients [women] if you don’t do that, they will just put you into trouble. Because when the baby is stuck there, it can’t breathe. But she would rather come and blame the midwife for not delivering the baby well. (Midwife 23)*

There is clear evidence of the use of violence – shouting, hitting and even beating– on labouring women to avoid auditing. Some midwives admitted being aggressive towards women, making the entire birthing atmosphere a rather dreadful one for women.

### Patriarchal pressures and ideologies

Healthcare systems and institutions often replicate the operations of society, including gender notions and norms. Therefore, how women are perceived and treated in society cannot be different from the kind of care they will receive in health facilities. Women’s positionality in society, patriarchal conceptualizations of motherhood, and notions about the female body and sexuality are replicated in the care provided in healthcare facilities.

#### Patriarchal notions of motherhood

The traditional conceptualization of motherhood, which denotes pain, suffering, sacrifices, and hard work is highly upheld by midwives. Common expressions such as ‘no pain no gain’ dominated the discourse and women who are unable to bear this perceived brutal pain of motherhood are rebuked. A woman narrating her childbirth experience recounted that:

*When I got there, they asked me to buy gloves so that they could examine me. Because it was my first vaginal examination, I was feeling so much pain and the midwife scolded me. She told me that is what they are going to be doing to me so if I’ll open my legs for them to do it, I should. As for that one, she scolded me a lot. (Woman 32)*

Another midwife emphasised:

*We expect them to abide by our rules, labour is very painful and so far as you have chosen to give birth by yourself it will not be easy. So, we expect that whatever we tell them they follow. Like to obey us. (Midwife 4)*

Also, a woman’s inability to endure the pain of motherhood is deemed as laziness and poor maternal effort, which must be corrected using violence. Based on this notion of motherhood, women are blamed for being the cause of their victimisation. Most midwives believe education of women on motherhood is the key to dealing with obstetric violence.

*Some of them are just too lazy and always complain that they are tired*. *(Midwife 18)*

*When you shout and hit the women, it puts some kind of fear in them to push. (Midwife 6)*

*Educating the women. If only the women would cooperate, definitely there wouldn’t be problems [obstetric violence]. So, it’s the pregnant women who need more education. (Midwife 4)*

#### Unequal power relations and dynamics

Healthcare professionals also uphold patriarchal notions about women being ignorant and incapable of making decisions over their bodies. This has created an atmosphere of control and power in the delivery room, where women’s agency is completely undermined based on this perceived ignorance. The concept of ‘obedience’, which demands subjectivity (from women) and superiority (healthcare workers) was greatly maintained by the midwives interviewed. As a result, maternity care is dominated by healthcare professionals’ opinions, wishes and ideologies as opposed to being women-centred. A woman narrating her birth experience revealed that:

*I observed the way the midwives talk to us and it makes me sad. Sometimes when we ask them questions, the way they answer will not be pleasing. Sometimes they make it look like you have not gone to school before and you don’t know what you are talking about. So frankly speaking, the way they handle us at the hospital I really don’t like it. (Mother 27)*

Some midwives justified unconsented episiotomies based on the perception that women are ignorant. A midwife recounted:

*We use our discretion because we have more knowledge in childbirth than the woman and so we know it’s best to perform such a surgical procedure so we just go ahead to do it. (Midwife 15)*

Sometimes a women’s request for the right care is perceived as audacious, insubordinate, or a challenge to the authority and knowledge of healthcare professionals. To maintain power, women are either threatened or forced to endure abuse. Women narrating their birth experiences described:

*After they performed an episiotomy and they were about to stitch it, I was feeling pain, so I asked them to inject me with anaesthesia. The midwife said ‘Whether she injects me or not, am I the one who will teach her how to do her job?’ And I said oh no, I’m feeling so much pain so I’m just begging her to inject me to reduce the pain I’m feeling. The way she behaved, it was as if I was dumb. (Mother 17)*

*They told me to lie on my left side but I was feeling pain, so I kept turning and one of the midwives scolded me. She said that if it becomes difficult, they will abandon me. (Mother 6)*

#### Controlling knowledge and bodies

The unequal power relations between women and healthcare professionals is sustained through the control of knowledge and women’s bodies. The withholding of information can be used to keep women in a position of subjugation to authority. Sometimes women are abandoned and even left to deliver without any assistance from health care professionals.

*When she told me to push, I asked her how I should push while trying it. She rebuked me and said ‘Is that how we do it? While you’re lying down like a log, is that how we do it?’ I asked her how I was supposed to do it [push] but she didn’t teach me. (Mother 11)*

*The next midwife that came, even when you are asking her a question, it is as if she doesn’t want to answer you. She doesn’t even recognize that you are a human being coming for help. So, when the head of the child was coming out, I called her but she told me ‘what is coming?’. When I called her, she didn’t mind me. I had to squat, I delivered on the floor and when she got there she was talking harshly. She asked me why I gave birth on the floor and I told her that when I was in labour I called her but she did not answer me. She said ‘If I didn’t answer you, that was not an excuse to give birth on the floor’*. *(Mother 8)*

#### Objectification of the female body

In patriarchal ideology, the female body is an object of sexualisation, mainly for a man’s pleasure. Although most midwives are women, they also hold these preconceptions and enforce them during care. Sutures following episiotomies or tears are conducted mainly for the sexual pleasure of men. A midwife’s response to women who reject sutures was:

*We tell the person that if we don’t stitch it, her perineum will become wide and her husband will not enjoy the sex because she didn’t allow us to do it. (Midwife 9)*

This was corroborated by another midwife who also emphasised that:

*If they object to it, we explain that if she does not allow me her husband cannot enjoy sex with her again. The place would be wide. (Midwife 16)*

From the above quotes, it is evident that the idea behind sutures is not to promote the well-being of women but rather to satisfy men’s sexual desires.

#### Gender preference

In a patriarchal society like Ghana, there is a general preference for male children, putting women under pressure. This pressure is even more profound with the emergence of health technology where the sex of a child can be determined at the early stages of pregnancy. Some women are even abandoned by their families in health facilities due to the sex of the baby. A midwife, narrating one of such events revealed:

*A man wanted a male child and it happened that the woman gave birth to a female child. Upon realizing that the baby was a girl, the man ran away and left the wife at the hospital. Therefore, I had to go to the Chief’s palace for him to be talked to before accepting them [woman and baby] in [the house]. (Midwife 4)*

Another midwife also stated:

*Mostly maybe the gender of the baby. Most Muslims value a male baby more than a female baby. Even last week we had a case here. The lady had a baby girl, she knew because the scan detected a baby girl but she lied to her husband that she would deliver a baby boy, so the husband was happy. When we delivered the baby girl, the woman turned her face away. Then we forced her to mention the gender. Later when the husband saw the baby, he was angry and said ‘No, this baby is not my baby’. The woman was the only one who confirmed that she lied to her husband. So, the gender of the baby leads clients to ignore their baby. They do not even want to pay; they want the baby to die before it comes. (Midwife 17)*

From these extracts, it is apparent that the detention of women in health facilities after childbirth is sometimes a coerced one. Sometimes women have to be kept in the hospitals because they are abandoned by their partners due to the sex of the child they delivered.

There was also evidence of discriminatory practices against single women in the data. In Ghana, there is a general stereotypical view that women who engage in premarital sex are promiscuous. Hence, discriminatory practices are ways of punishing such women, in order to uphold societal values. In particular, married women are given preferential treatment, especially when they are accompanied by their husbands for obstetric services.

*When you are pregnant and you come to the hospital with your husband you do not join a queue. At the hospital, that is what I saw. You do not join the queue, so a person can be at the hospital, maybe from morning to afternoon because the queue is long, and someone will come with the husband, they will just go straight to the married woman, they will perform the examination and she is gone. Therefore, if you come to the hospital with your husband, you don’t join the queue. (Mother 25)*

### Gender insensitivity

The importance a society places on mothering and childbirth can sometimes be represented through the financial resources and investment given to maternity care. How the resources are tailored towards meeting women’s specific needs can be even more important. Gender insensitivity in resource provision could portray how women’s needs and rights are undermined.

#### Gender insensitive infrastructure

In Ghana, this study revealed that the infrastructure and resources provided for maternity care were gender insensitive. The maternity wards are built in a similar pattern as all other wards. They are without the demarcations needed to protect the privacy women need for childbirth. Due to this gender-insensitive infrastructure, women are subjected to non-confidential care, undermining women’s bodily integrity and their psychological well-being. Women’s right to birth companions is also denied as a result of infrastructural deficits.

*You know our setting; we don’t have any privacy. But if we had curtains, their companions might be allowed in. But looking at our setting, it’s not allowed because other women have undressed and so we can’t allow someone’s husband to come in just to provide support for his wife. (Midwife18)*

*In as much as we want the husbands to be involved, we try to ensure privacy for the other women. In the same way, my husband wouldn’t want any other man to see me naked, we also don’t want other men to invade the privacy of another man’s wife. The men would have been allowed to stay with their wives if there were cubicles for the women. (Midwife 29)*

#### Medical system’s model of care

The study also found the medical system’s model of care to be problematic. This model emphasises the medical aspect of care, neglecting the social aspect. The medicalization of childbirth therefore means a drastic reduction in the time midwives spend with the women, making them unable to provide the physical, psychological and emotional support that women need. Even more, a women’s right to information regarding their bodies is often overruled due to the reduced time available for midwives to care for the women. Women are discharged only six hours after childbirth, which midwives deemed too short for adequate postpartum care. Some midwives explained:

*We wish we could give some sacral massages to our clients. With every examination we do, we are supposed to sit our client down and explain in detail where the progress has reached and all those things. But we just mention to her, ‘Madam your cervix has dilated’, but in actual fact, it is not only the cervix we should talk about, there are other things we need to talk about. (Midwife 20)*

*It’s the infrastructure issues that made me mention that our labour ward is too small for us, so it shortens the time that we need to keep clients here and monitor them after delivery*. *(Midwife 20)*

These extracts from the data point to the systemic nature of obstetric violence. The health system’s model of care contradicts women-centred care, working against women rather than in favour of those it is supposed to care for.

## Discussion

This article examined how gender inequalities in health systems and the medical profession contributed to women’s experiences of obstetric violence in Ghana. The study revealed that the medical profession is very hierarchical and this results in power dynamics that put women at the lower cadres of the profession. This has implications for the working conditions of midwives. There are limited opportunities for career advancement and in-service training on contemporary delivery skills. This has created a skill and knowledge gap, which affects the kind of support they give and the skills they need to ensure a violence-free delivery. In addition, midwives denied women their preferred birthing positions based on the notion that the lithotomy position is best for managing complications. However, research has established that the lithotomy position is rather closely associated with birth complications- “increased need for episiotomy and increased chance of forceps delivery or caesarean section” [44]. Thus, the lack of training puts women at risk.

Previous studies have demonstrated that the rate of maternal and neonatal deaths can be significantly reduced if midwives receive adequate and continuous in-service training [45, 46]. The researchers particularly emphasised that up to 4.3 million lives annually could be saved if more investment was made in the midwifery profession; yet, many countries do not prioritise the of midwives. As demonstrated above, the lack of training leads to obstetric violence which impacts women’s physical and mental health.

This research has also outlined how the huge pay gap and the patient-carer ratio between midwives and medical doctors is a clear by-product of the patriarchal and gender inequality perpetrated in the health system. The comparatively low salaries of midwives have been recorded in many countries including the United Kingdom, USA, New Zealand, and Netherlands [47–50]. Filby et al. [51] found that one-fifth of midwives in Africa, Asia and the Americas depended on secondary sources of income for survival due to poor salaries. In this study, there was evidence of neglect, extortion and detention of women due to the trading activities that midwives engaged in to supplement their salaries. With a high patient-midwife ratio, the midwives work in an atmosphere of stress and burnout which leads to poor care, neglect, and abuse in the delivery room. Caring for labouring women carries an additional emotional burden, and an unhealthy state of mind has negative consequences on care-seekers. The findings of this study are consistent with Boakye et al.’s [52] study in Ghana, where the shortage of nurses and midwives created an overwhelming workload which impacted their capacity to sustain their values and perform their responsibilities. The verbal abuse and shouting are indicators of midwives’ inability to cope with the stress [25]. Also, Hall [53] and Deery [54] have demonstrated in their studies that stress and burnout of midwives and nurses reduce their compassion in the care they provide.

Midwifery is considered an extension of women’s traditional care role, which has led to the the profession being demeaned and devalued [51]. Moreover, because women and not men give birth, the general attitude towards midwifery is equivalent to the general perception and position of women within a patriarchal society. Compared to other sections of the healthcare system, they are severely under-resourced, less respected and blamed for negative birth outcomes. Poor perceptions of midwives also are held in Europe where midwives are labelled ‘half-taught’ or ‘totally ignorant’ [55]. Evidence in this study revealed that such perceptions have a negative impact on the care provided and contribute to women’s experiences of obstetric violence.

There is also a strong correlation between the lack of resources and obstetric violence. For example, the midwives neglected women’s requests for care when they perceived the lack of equipment threatened their health, especially in cases with HIV-positive women. Previous studies on midwifery in Ghana have established the acute lack of resources as an everyday reality which led to dehumanized care, with midwives working in an uninhabitable environment [34, 52]. In addition, the disrespect of midwives caused a transfer of aggression, leading to poor treatment of women. The findings of this study corroborate Filby et al.’s results [51], where the researchers also found that 36% of midwives admitted being disrespected while 37% were bullied by senior medical staff. Although sanctions for medical negligence are crucial, an analysis of the accountability demands for midwives (feminised) and medical doctors (masculinised) in Ghana indicates structural inequalities. Unlike midwives who are subjected to ‘auditing’ by health authorities for all cases of unsuccessful delivery, the Ghanaian Criminal and Other Offences Act stipulates that medical doctors can only be sanctioned when evidence of intentional negligence in unsuccessful surgeries can be proven by the family of the deceased before the court of law [56]. The ambiguity of the law in defining ‘intentional/unintentional negligence’ and the alienation of the medical authority in the sanctioning process makes it difficult for medical doctors to be held accountable. The gender inequality and the hierarchical nature of the medical profession determine women’s childbirth experiences. In this study, the incessant blaming of midwives places them under higher pressure. In order to be successful and avoid ‘auditing’, midwives become aggressive and violent in their approach towards childbirth, leading to more violent deliveries.

This study also revealed how patriarchal ideologies and pressures in society as a whole combine to make women extremely vulnerable to abuse. Health institutions replicate how society operates. Therefore, patriarchal notions of motherhood, which connote pain, sacrifice, and hard work are also upheld by midwives; and, women who fall below this expectation are abused. Women are also denied agency over their bodies and the right to consented care due to the perception that they are ignorant. Feminist scholars contend that the “aetiology of dominance is based on the patriarchal structure of society that is built on male superiority, female subordination, and sex-stereotyped roles and expectations’’ [57–59]. Hence, women cannot be expected to wield any power in the delivery room when they have no such power in society. Violence thrives in highly hierarchical power relations and atmosphere.

In the case of maternity care, childbirth places women in a uniquely vulnerable position. In this study, withholding knowledge, controlling bodies, abandonment, and dehumanisation of women are major instruments of domination and control. According to Goodyear-Smith and Buetow [60], the relationship between patients and caregivers is characterised by “unequal power dynamics where the caregiver holds the knowledge and authority to the detriment of the patient, who is placed in a vulnerable position’’. When gender intersects with care-existing vulnerabilities, violence becomes inevitable. The sexualisation of the female body manifested itself in notions behind stitches after episiotomies or tears, where men’s sexual pleasure is prioritised. Also termed as the “husband’s stitch” or “daddy stitch”, this invasive practice is an unjustifiable control over women’s bodies, intended to cater for a more male-centric interest. Other studies have reported similar practices in Brazil and Greece [61, 62] with women equating their experiences to Female Genital Mutilation. Experts have declared this invasive procedure as medically unnecessary and potentially harmful [63], yet many healthcare professionals continue such practices even without asking for women’s consent. Gender insensitivity in resource provision and medical systems model of care emerged as crucial contributory factors of obstetric violence. The open maternity wards deprived women of confidential care and birth companions, which are critical for childbirth. Studies have demonstrated that the odds of obstetric violence are drastically reduced (by 75%) with the presence of birth companions [64, 65]. Additionally, the medical system’s model of care emphasises medicalisation and completely undervalues the social aspect of care. Among the outcomes are depersonalised and hurried care, with severe consequences for the quality of obstetric health care provision.

## Conclusion

In conclusion, this study highlights the gendered dynamics of obstetric violence. The picture emerging from the study demonstrates that gender inequality is embedded within health systems and contributes significantly to obstetric violence in Ghana. Lack of training opportunities for midwives, heavy workload, poor working conditions and wages, lack of respect and resources, and unequal demands and sanctions are manifestations of structural and systemic inequalities, which lead to the poor and abusive treatment of labouring women.

The study also revealed that the patriarchal conceptualisation of women and their bodies further put women in a vulnerable position for abuse. This study points to the need for the empowerment of women and girls regarding their autonomy over their bodies, decision making rights and sexualities. The deconstruction of the gendered hierarchical system in the healthcare system and profession needs to be addressed urgently. Additionally, this study points to the need for continuous retraining of professional midwives, particularly on humanised birthing practices. More crucially, as poor working conditions and the high midwife-patient ratio compromise the quality of care, improving the working conditions of midwives and recruiting more midwives is paramount.

One of the crucial findings of this study is that gender inequality in society has a great impact on how healthcare professionals perceive and treat women. In view of this, obstetric violence interventions should focus on structural changes that aim to transform discriminatory gender norms, stereotypes, and underlying sociocultural factors that shape obstetric violence. The involvement of men in women’s reproductive care is also very crucial. The training of midwives and all health professionals on gender equality is key. From this study, it is clear that the medical system’s model of care has dire consequences for women. Therefore, the incorporation of a social model of care into obstetric care is crucial.

## Limitations

Although the study provides great insight into gender and obstetric violence, it was conducted in only two of the sixteen regions in Ghana and relied solely on qualitative interviews involving 65 interviewees. This limits the generalisability of the results. It must however be stated that Ghana’s healthcare system is centralised, with midwives trained in different parts of the country. Hence, similar practices will likely be found in different regions of Ghana. Also, because the interviews with midwives were conducted at health facilities, social desirability bias might have occurred, which could have resulted in an underreporting of their experiences. While studies of this nature, where women describe their past birth experience, could be impacted by recall bias, previous studies have demonstrated that childbirth memories last up to 20 years [66, 67]. Therefore, the findings of this study are unlikely to be affected by recall bias.

## Data Availability

The data presented in this study are available on request from the corresponding author. The data are not publicly available due to the ethical principles of the University of Konstanz and the Ghana Health Service Ethics Committee, which guided this study.
